# Arrhythmogenic Right Ventricular Cardiomyopathy in a Patient Experiencing Out-of-Hospital Ventricular Fibrillation Arrest Twice: Case Report and Review of the Literature

**DOI:** 10.7759/cureus.21457

**Published:** 2022-01-20

**Authors:** Zahid Khan, Mohammed Abumedian, Yousif Yousif, Animesh Gupta, Su L Myo, Gideon Mlawa

**Affiliations:** 1 Cardiology, Royal Free Hospital, London, GBR; 2 Geriatrics, Barking, Havering and Redbridge University Hospitals NHS Trust, London, GBR; 3 Internal Medicine, Barking, Havering and Redbridge University Hospitals NHS Trust, London, GBR; 4 Acute Internal Medicine, Barking, Havering and Redbridge University Hospitals NHS Trust, Romford, GBR; 5 Cardiology, Barking, Havering and Redbridge University Hospitals NHS Trust, London, GBR; 6 Internal Medicine, Diabetes and Endocrinology, Barking, Havering and Redbridge University Hospitals NHS Trust, London, GBR

**Keywords:** sudden cardiac arrest, ventricular arrhythmia, right ventricular failure, arvc, ventricular dysrhythmia, implantable-cardioverter defibrillator

## Abstract

We present a case of a 62-year-old male who was admitted to the hospital with out-of-hospital ventricular fibrillation (VF) arrest. He had a VF arrest in 2011 and was admitted to another hospital. He had several investigations excluding cardiac magnetic resonance imaging, all of which were normal. He was playing tennis on both occasions when he experienced the VF arrest. His electrocardiogram on admission showed AF with partial right bundle branch block, inverted T waves in V1-V2, low voltage QRS complexes, ventricular ectopic in lead V1-V2, and prolonged QTc. His echocardiogram showed normal left ventricular function and a dilated right ventricle. Cardiac magnetic resonance imaging showed a dilated RV cavity size with impaired systolic function and dyskinetic region in the mid-ventricular free wall proximal to the insertion of the moderator band and late gadolinium enhancement in both right and left ventricles insertion points and mid-wall late gadolinium enhancement in the basal inferolateral wall suggestive of arrhythmogenic right ventricular cardiomyopathy. He had a single chamber VVI implantable cardioverter-defibrillator fitted for primary prevention and was discharged home. He had outpatient follow-up and showed good improvement and his implantable cardioverter-defibrillator checks were satisfactory and did not experience any shocks.

## Introduction

Arrhythmogenic right ventricular cardiomyopathy (ARVC) was first described by Frank and Fontain in 1978 based on findings in six patients presenting with ventricular tachycardia [1]. They described ARVC as a “total or partial replacement of right ventricular muscle with fibro-fatty tissues associated with left bundle branch block morphology arrhythmia” [1]. ARVC is an inherited condition in which the right ventricle and sub-epicardial region of the left ventricle are replaced by fibrofatty tissue [1,2]. This results in tachyarrhythmias and is more common in young individuals and athletes. The diagnosis of ARVC is challenging due to varied and non-specific presentations and it accounts for sudden death in 30% of young adults and in 5% of those less than 65 years of age [1,2]. ARVC is the most common cause of death in young patients following hypertrophic obstructive cardiomyopathy [2]. It is increasingly recognized in forensic practice as most cases are diagnosed following death [3]. Most young patients present with sustained ventricular tachycardia with left bundle branch block morphology, sudden death, or isolated right or biventricular heart failure [4]. Right ventricular thrombus is also common in patients with ARVC [5]. Several case reports have been published previously and most are young patients, with only a few case reports involving patients more than 60 years of age. Our patient was 62 years old and presented with VF arrest on two occasions.

## Case presentation

A 62-year-old man had sudden-onset ventricular fibrillation (VF) induced cardiac arrest whilst he was playing tennis. He had bystander cardiopulmonary resuscitation (CPR) for 10 minutes later and was given three shocks by the London ambulance services team (LAS) for ventricular fibrillation. The patient had a return of spontaneous circulation (ROSC) and was admitted to the intensive care unit (ITU) following intubation in the emergency department. He had a similar VF cardiac arrest 10 years ago while playing tennis and was admitted to another hospital, however, we did not have access to his medical records from that admission. Based on the patient information, he had several investigations including normal echocardiography and coronary angiogram, and the cause for his VF arrest was not identified. He however did not have cardiac magnetic resonance imaging (CMR). According to the patient, an implantable cardioverter-defibrillator was not offered to him during his previous admission in view of no ischaemic cause for the VF arrest.

**Figure 1:**
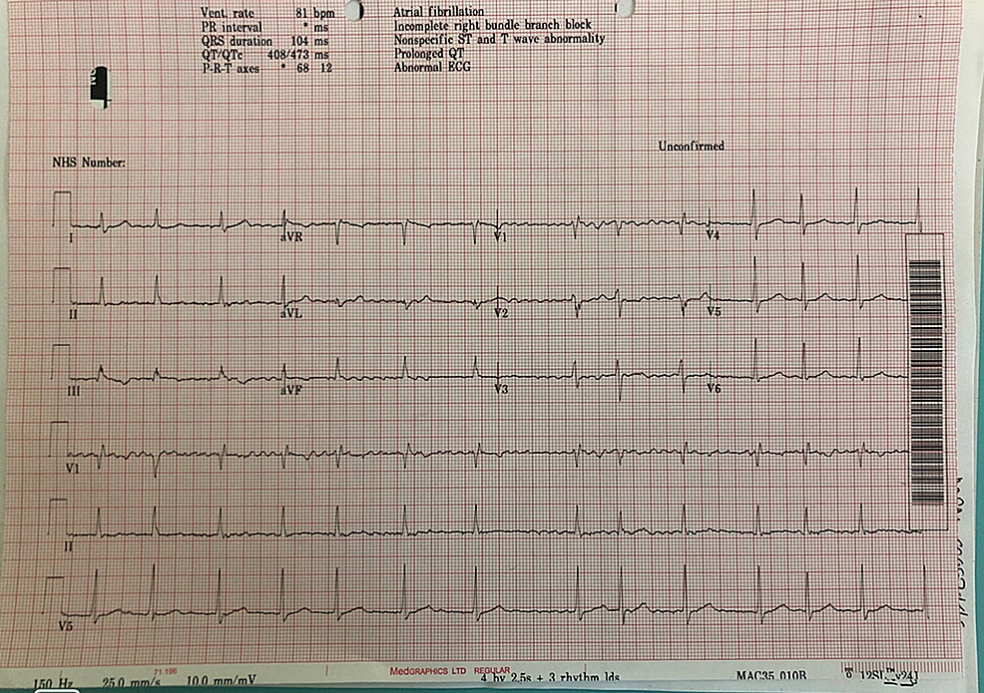
Electrocardiogram showing atrial fibrillation

His current admission electrocardiogram (ECG) showed AF with good rate control, partial or incomplete right bundle branch block (RBBB), and prolonged QTc of 473 milliseconds (Figure 1), and paramedics ECG showed VF.

His lab tests results are shown below in Table 1.

**Table 1:** Bloods result for this patient on day of admission

Blood test	Result	Normal value
Haemoglobin	135	133-173 g/L
White cell count	16.88 X10*9/L	3.8 - 11 X10*9/L
Neutrophil	13.93 X10*9/L	2 - 7.5 X10*9/L
pro-brain natriuretic peptide (BNP)	656	100-300 pg/mL
Glycated haemoglobin test	37 mmol/mol	48 mmol/mol
Troponin T	135	0 – 13 ng/L
Repeat Troponin T	277	0 – 13 ng/L
Sodium	138	133-145 mmol/L
Potassium	4.6	3.5-5.3 mmol/L
Urea	5.7	2.5-7.8 umol/L
Creatinine	94	59 – 120 mL/min

**Figure 2:**
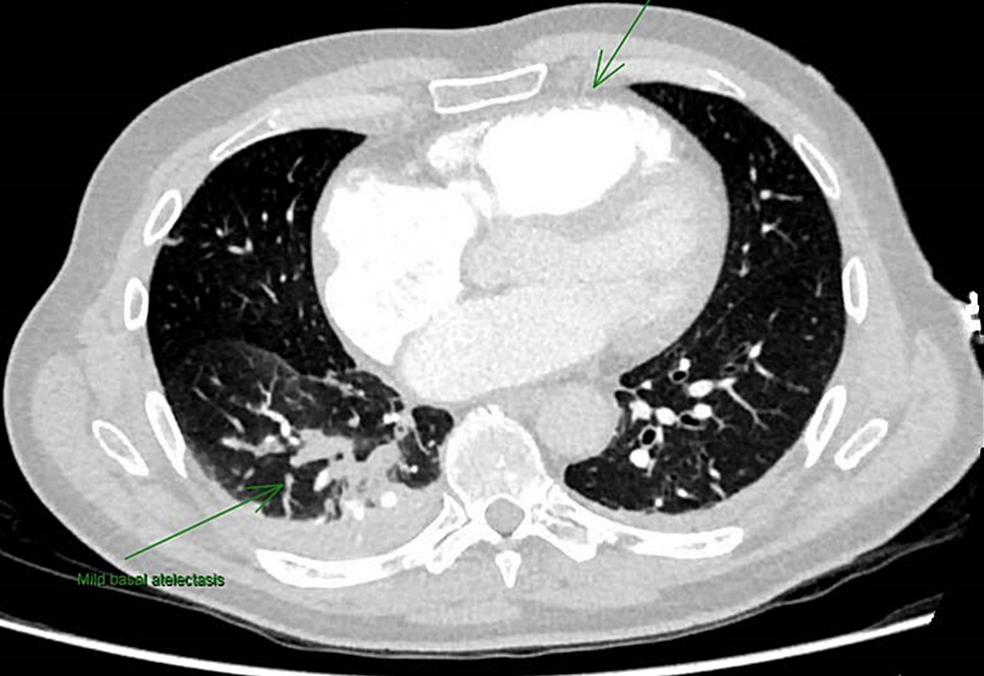
Computerized tomographic pulmonary angiogram showing dilated right ventricle and atria with atelectasis.

He underwent an emergency coronary angiogram which showed normal coronary arteries. He had a computerised tomographic pulmonary angiogram (CTPA) that showed enlarged right ventricle, bi-basal consolidation, and broken anterior ribs likely due to cardiopulmonary resuscitation (CPR) (Figure 2).

**Figure 3:**
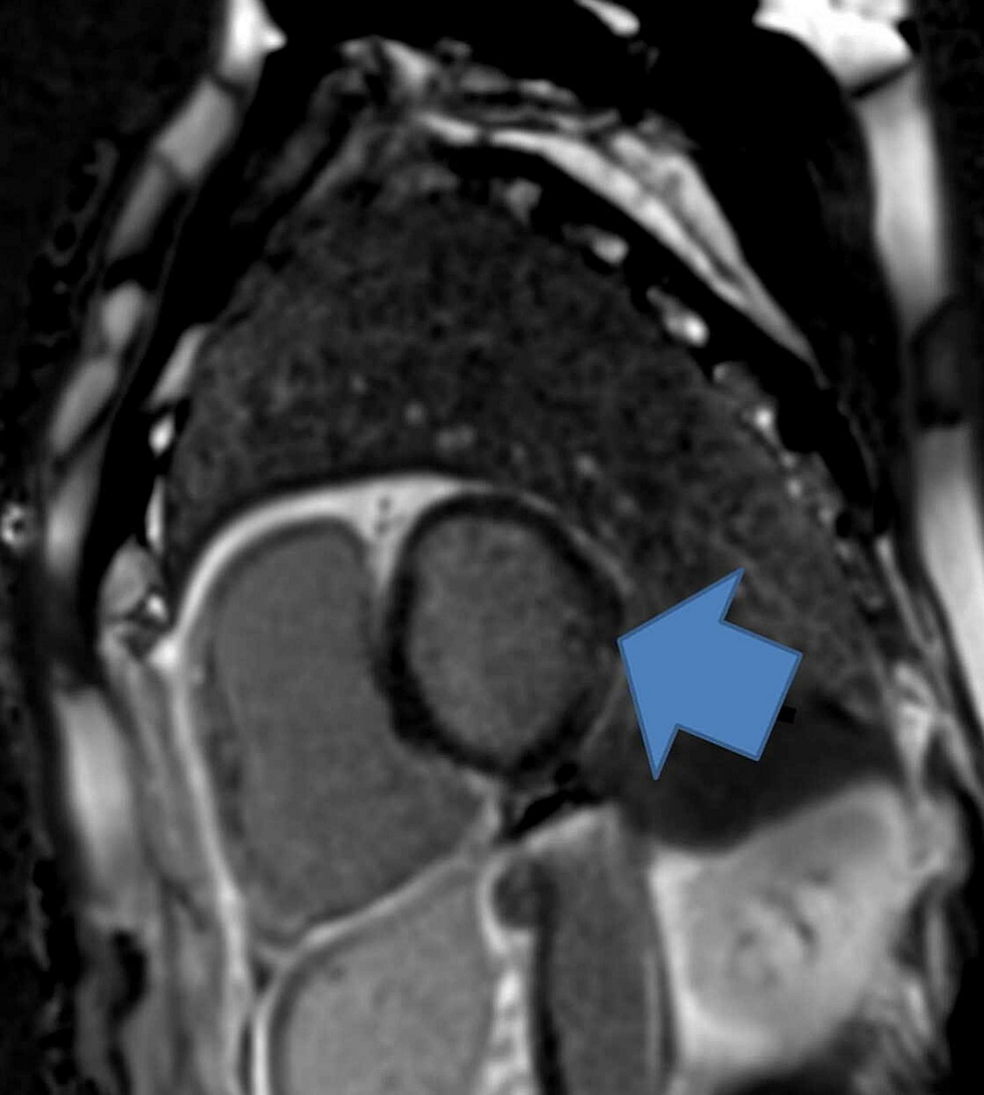
CMR showing features of ARVC such as LGE (blue arrow) Arrhythmogenic right ventricular cardiomyopathy (ARVC); non-specific late gadolinium enhancement (LGE)

**Figure 4:**
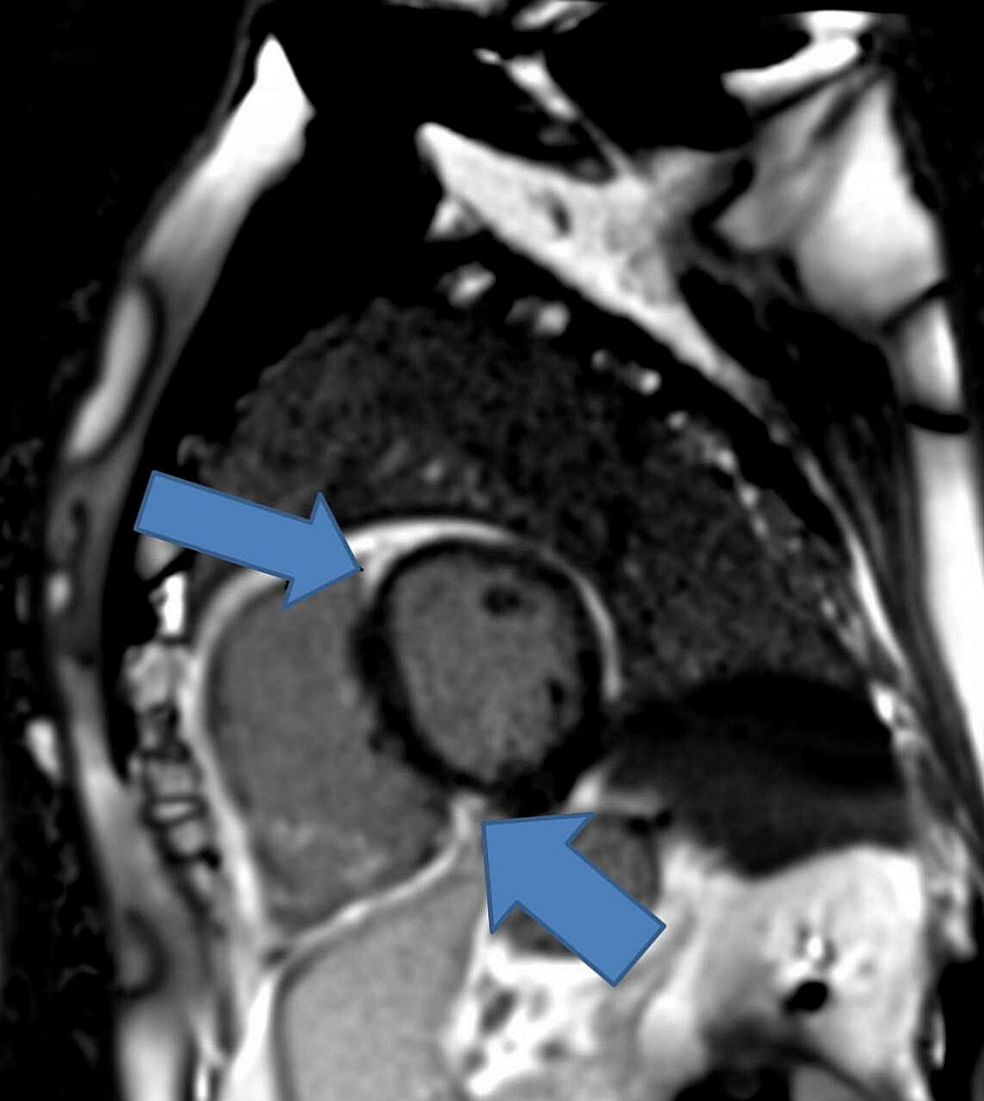
CMR showing LGE, a feature of ARVC (blue arrows) Arrhythmogenic right ventricular cardiomyopathy (ARVC); non-specific late gadolinium enhancement (LGE); cardiac magnetic resonance imaging (CMR)

**Figure 5:**
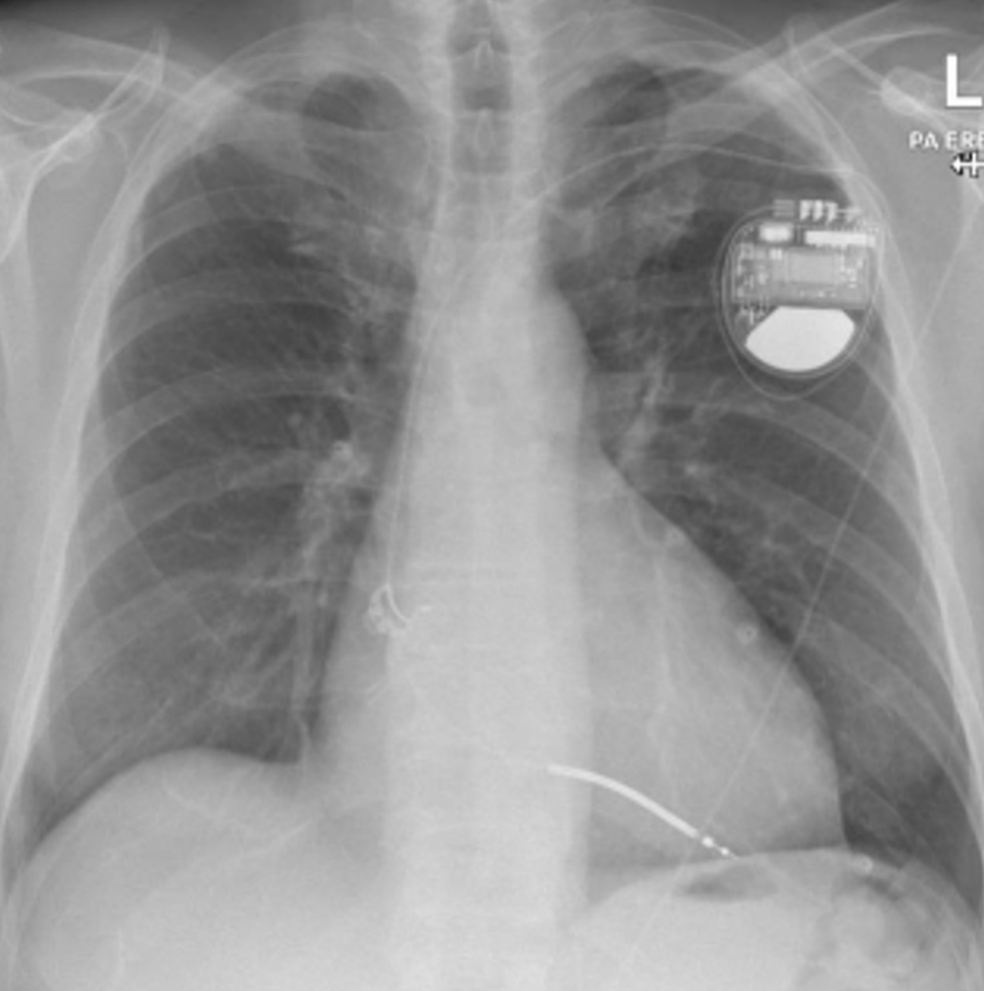
Chest X-ray showing single-chamber implantable cardioverter-defibrillator (ICD)

Following this, he had a repeat echocardiogram two weeks later that showed good LV function of about 60%, bi-atrial dilatation, dilated RV, and moderate tricuspid regurgitation (TR). He had a ventilation/perfusion scan in view of his enlarged right ventricle that did not show any pulmonary embolism (PE). He had CMR following this to assess for ARVC which showed dilated and impaired RV with dyskinesia of the mid-ventricular segment of the RV free wall (Figures 3-4). The LV function is low-normal with a non-specific late gadolinium enhancement (LGE) in the lateral wall what could reflect biventricular involvement. He had a cardiac septal biopsy that showed non-inflamed myocardium with focal subendocardial fibrosis with no morphological features of ARVC or sarcoidosis. This patient met four major criteria for ARVC including ventricular arrhythmia, fibrofatty replacement of the myocardium on tissue biopsy, LGE in RV, and global RV dysfunction. He was discussed in an electrophysiology multidisciplinary meeting (MDT) and had VVI ICD fitted prior to discharge without any complications as shown on the chest x-ray post-ICD implantation (Figure 5).

He remained in permanent AF and was anticoagulated. He attended outpatient follow-up and ICD check that was satisfactory and has made a good recovery since.

## Discussion

Hypertrophic heart disease is followed by ARVC as the second-most common cause of death in young patients and it can also present in middle-aged patients. As the disease is more common in young adults and adolescents who may present with palpitations, fatigue, or syncope episode, it is important for paediatricians to be aware of this condition as young patients can face sudden unexpected death from the disease. Marcus et al. in 1982 described the disease as ARVD as they believed the disease to be caused by congenital weakness in the development of the right ventricle [6]. Thiene et al. in 1988 reported a series of cases of sudden cardiac deaths in young adults on exertion, and the autopsy findings in these patients were suggestive of ARVD [7]. Electrocardiograms showed right precordial leads with negative T waves and left bundle branch (LBBB) morphology type ventricular arrhythmia [6,7].

The true incidence of ARVC is unknown; however, the prevalence amongst the general population is approximately 0.02-0.1% [8]. The prevalence of ARVC shows significant geographical variation and it accounts for sudden death in 5% of patients under 65 years of age and 3-4% of young athletes in the United States. It affects males more than females and the disease is more common in young patients. About 80% of cases occur in young patients < 40 years of age, therefore it should be considered in young adults or athletes presenting with a history of syncope, arrhythmias, and/or cardiac arrest.

Several case reports of ARVC causing sudden death in young patients have been published over the last two decades and most of these patients were diagnosed on post-mortem biopsies. Choung et al. (2017) reported a case of a 26-year-old east African professional athlete who was 36-37 weeks pregnant, suffering a cardiac arrest in a restaurant, and had a history of previous cardiac arrest. She had been diagnosed with right ventricular outflow tract-induced ventricular tachycardia in the past which was associated with her previous cardiac arrest and underwent ablation. She had unsuccessful resuscitation and autopsy findings showed a focal area of myocardial thinning at the right ventricular apex, and histology of the cardiac biopsy confirmed ARVC findings such as myocardial cell degeneration and replacement by adipose tissue [9]. Low et al. (2020) published a case report of a 20-year-old patient who collapsed during a futsal match and was diagnosed with ARVC and right ventricular thrombus on CMR [10].

Wu et al. (2021) published a case report of a 43-year-old patient with three syncopal episodes during exercise and was found to be in VT in the emergency department and was administered intravenous propafenone to terminate his VT [11]. His repeat ECG post-VT termination showed normal sinus rhythm with negative T waves and a delayed S-wave upstroke from leads V1 to V4. His CMR showed RV dilatation and free wall thinning; regional RV akinesia, fibrofatty infiltration, and impaired RV function. He had multiple inducible ventricular tachycardias and voltage mapping demonstrated scar tissue in the right ventricular anterior and posterior walls. He was diagnosed with ARVC and received treatment with combined catheter ablation to both endocardial and epicardial walls to achieve a satisfactory outcome [11].

ARVC should be always be considered as the most likely diagnosis in patients with ECG and overt structural changes. It has four particular phases consisting of concealed phase, apparent electric disorder, right ventricular failure, and the advanced phase with severe diffuse bi-ventricular failure resembling dilated cardiomyopathy [12]. The concealed phase is the first phase and often precedes the other three phases associated with a high arrhythmia burden and increased risk of Sudden cardiac death (SCD). Patients may present with symptoms such as intermittent chest pain and are usually diagnosed with myocarditis due to raised troponin and normal coronary angiogram [12].

There is a lack of gold-standard tests or pathognomonic criteria for making a definitive diagnosis of ARVC. The revised 2020 Task Force Criteria for diagnosis of ARVC is based on the presence of major criteria and minor criteria (Table 2) [12].

**Table 2:** Summary of Changes in the 2020 International Criteria for Diagnosis of ARVC Arrhythmogenic right ventricular cardiomyopathy [13].

Summary of Changes in the 2020 International Criteria for Diagnosis of ARVC		
I. Global or regional dysfunction and structural alteration	RV WMA plus dilatation/dysfunction RV WMA in isolation	Modified in major criterion only; reference values of EDV and EF modified according to the imaging test specific monograms for age, sex, and BSA New minor criterion
II. Tissue characterization	Fibrofatty myocardial replacement on EMB RV LGE on CMR	Modified in major criterion only New major criterion
III. Repolarization abnormalities	Inverted T waves in right precordial leads	Unchanged
IV. Depolarization and conduction abnormalities	Epsilon waves Late potentials by SAECG QRS terminal activation delay in right precordial leads	Downgraded to minor criterion Not included Unchanged
V. Arrhythmias	Nonsustained and sustained VT with LBBB morphology Frequent ventricular extrasystoles (>500 per 24 h)	Unchanged Modified in major or minor criterion according to the morphology of the ectopic QRS
VI. Family history/genetics	Clinical or pathological diagnosis in a first‐degree relative Identification of a pathogenic or likely pathogenetic mutation History of either suspected ARVC or premature SCD caused by suspected ARVC in a first‐degree relative Clinical or pathological diagnosis in a second‐degree relative	Unchanged (major) Unchanged (major) Unchanged (minor) Unchanged (minor)

**Figure 6:**
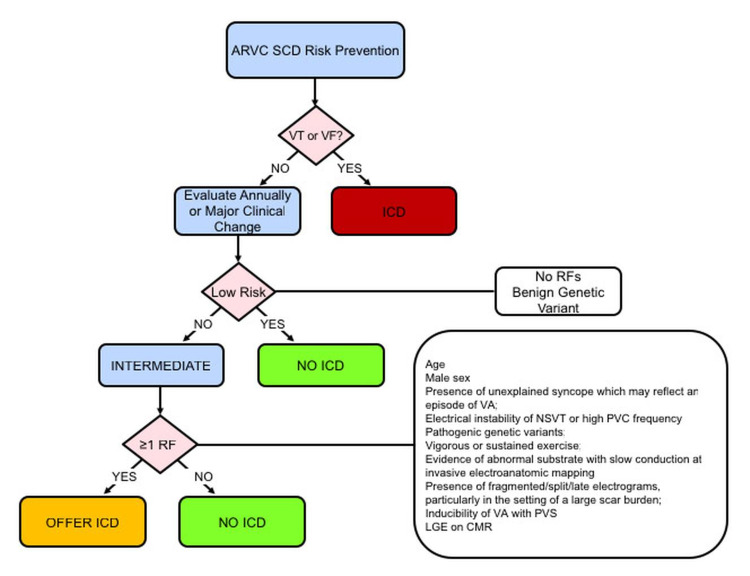
Flow chart below provides key risks and indications for ICD implantation Source: American College of Cardiology. Risk stratification in arrhythmogenic RV cardiomyopathy/dysplasia without an ICD [16].

Each major criteria is scored two points and each minor criteria is scored one; at least two major criteria or one major and two minor criteria, or four minor criteria should be present to give a score of four to make a diagnosis of ARVC. The diagnosis of ARVC is considered to be borderline in patients with a score of three points [12]. The 2015 International Task Force Consensus Statement on risk stratification recommends ICD implantation for secondary prevention in all high-risk patients including those with sustained ventricular tachycardia and aborted sudden cardiac death. It also recommends ICD implantation for primary prevention in patients with severe uni-ventricular or bi-ventricular dysfunction [14,15]. Male sex and exercise were risk factors for SCD or aborted sudden cardiac death and a predictor for ICD implantation besides other risk factors [14,15]. The flow chart below provides key risks and indications for ICD implantation (Figure 6).

## Conclusions

In summary, our case involved a 62-year-old male patient who had two out-of-hospital ventricular fibrillation cardiac arrests and was successfully resuscitated both times. The results of his coronary angiogram were normal and cardiac magnetic resonance imaging features were suggestive of the diagnosis of arrhythmogenic right ventricular cardiomyopathy and his cardiac biopsy revealed subendocardial fibrosis. He had an implantable cardioverter-defibrillator implanted to minimize the risk of future sudden cardiac death.

Arrhythmogenic right ventricular cardiomyopathy is a rare inherited cardiac muscle disease and should be considered as a possible cause in young patients presenting with ventricular tachyarrhythmias as they are at risk of sudden death if not appropriately managed. It is important for physicians to be aware of the possibility of arrhythmogenic right ventricular cardiomyopathy in young adults who may present with palpitations, fatigue, and syncope or exercise-induced cardiac arrest. Making a diagnosis of arrhythmogenic right ventricular cardiomyopathy can be difficult and patients with ARVC should be offered implantable cardioverter-defibrillator for primary prevention to prevent sudden death. These patients may have right ventricular dilatation alone or biventricular dilatation. 
